# Supplementary material for: P-TRAP: a Panicle Trait Phenotyping tool
Source: BMC Plant Biol. 2013 Aug 29;13:122. doi: 10.1186/1471-2229-13-122 (PMC3848748; doi:10.1186/1471-2229-13-122)
Supplement: Additional file 7 — P-TRAP source code. The Netbeans project that contains the source code of the application. [file 1471-2229-13-122-S7.zip › javadoc.zip!/javadoc/org-biolag-particleprocessor/org/biolag/particleprocessor/ParticleFinder.html]

ParticleFinder (ParticleProcessor)


JavaScript is disabled on your browser.


- Package
- Class
- Tree
- Deprecated
- Index
- Help

*org.biolag.particleprocessor 1.0*

- Prev Class
- Next Class

- Frames
- No Frames

- All Classes

- Summary:
- Nested |
- Field |
- Constr |
- Method

- Detail:
- Field |
- Constr |
- Method


org.biolag.particleprocessor

## Class ParticleFinder

- java.lang.Object
- - org.biolag.particleprocessor.ParticleFinder

- ---

    

  ```
  public class ParticleFinder
  extends java.lang.Object
  ```

  The detector of the particles in binary images.

  Author:
  :   faroq

- - ### Constructor Summary

    Constructors

    Constructor and Description

    `ParticleFinder()`
  - ### Method Summary

    Methods

    | Modifier and Type | Method and Description |
    | --- | --- |
    | `org.bioalg.imageprocessor.AImage` | `findParticles(org.bioalg.imageprocessor.AImage aImage, int minimumsize)` Find all particles in the input images given a minimum threshold size. |
    | `java.util.ArrayList<Particle>` | `getParticles()` |
    | `void` | `setParticles(java.util.ArrayList<Particle> particles)` |
    | `ParticlesModel` | `toParticlesModel()` Convert the particles to a `ParticlesModel` |

    - ### Methods inherited from class java.lang.Object

      `clone, equals, finalize, getClass, hashCode, notify, notifyAll, toString, wait, wait, wait`

- - ### Constructor Detail


    - #### ParticleFinder

      ```
      public ParticleFinder()
      ```
  - ### Method Detail


    - #### getParticles

      ```
      public java.util.ArrayList<Particle> getParticles()
      ```


    - #### setParticles

      ```
      public void setParticles(java.util.ArrayList<Particle> particles)
      ```


    - #### findParticles

      ```
      public org.bioalg.imageprocessor.AImage findParticles(org.bioalg.imageprocessor.AImage aImage,
                                                   int minimumsize)
      ```

      Find all particles in the input images given a minimum threshold size.

      Parameters:
      :   `aImage` - the input image
      :   `minimumsize` - the minimum size of pixels to consider as particle.

      Returns:
      :   An `AImage` object where the particles are labeled.

      See Also:
      :   `AImage.getconnectedObject(java.awt.Point, java.awt.Point, int, int)`


    - #### toParticlesModel

      ```
      public ParticlesModel toParticlesModel()
      ```

      Convert the particles to a `ParticlesModel`

      Returns:
      :   an object of `ParticlesModel`


- Package
- Class
- Tree
- Deprecated
- Index
- Help

*org.biolag.particleprocessor 1.0*

- Prev Class
- Next Class

- Frames
- No Frames

- All Classes

- Summary:
- Nested |
- Field |
- Constr |
- Method

- Detail:
- Field |
- Constr |
- Method
